# Western Diet Chow Consumption in Rats Induces Striatal Neuronal Activation While Reducing Dopamine Levels without Affecting Spatial Memory in the Radial Arm Maze

**DOI:** 10.3389/fnbeh.2017.00022

**Published:** 2017-02-09

**Authors:** Jason C. D. Nguyen, Saher F. Ali, Sepideh Kosari, Owen L. Woodman, Sarah J. Spencer, A. Simon Killcross, Trisha A. Jenkins

**Affiliations:** ^1^School of Health and Biomedical Sciences, RMIT UniversityBundoora, VIC, Australia; ^2^School of Psychology, University of New South WalesKensington, NSW, Australia

**Keywords:** western diet, high fat diet, neuronal activation, spatial memory, cognition, striatum, dopamine

## Abstract

Rats fed high fat diets have been shown to be impaired in hippocampal-dependent behavioral tasks, such as spatial recognition in the Y-maze and reference memory in the Morris water maze (MWM). It is clear from previous studies, however, that motivation and reward factor into the memory deficits associated with obesity and high-fat diet consumption, and that the prefrontal cortex and striatum and neurotransmitter dopamine play important roles in cognitive performance. In this series of studies we extend our research to investigate the effect of a high fat diet on striatal neurochemistry and performance in the delayed spatial win-shift radial arm maze task, a paradigm highly reliant on dopamine-rich brain regions, such as the striatum after high fat diet consumption. Memory performance, neuronal activation and brain dopaminergic levels were compared in rats fed a “Western” (21% fat, 0.15% cholesterol) chow diet compared to normal diet (6% fat, 0.15% cholesterol)-fed controls. Twelve weeks of dietary manipulation produced an increase in weight in western diet-fed rats, but did not affect learning and performance in the delayed spatial win-shift radial arm maze task. Concurrently, there was an observed decrease in dopamine levels in the striatum and a reduction of dopamine turnover in the hippocampus in western diet-fed rats. In a separate cohort of rats Fos levels were measured after rats had been placed in a novel arena and allowed to explore freely. In normal rats, this exposure to a unique environment did not affect neuronal activation. In contrast, rats fed a western diet were found to have significantly increased Fos expression in the striatum, but not prefrontal cortex or hippocampus. Our study demonstrates that while western diet consumption in rats produces weight gain and brain neuronal and neurotransmitter changes, it did not affect performance in the delayed spatial win-shift paradigm in the radial arm maze. We conclude that modeling the cognitive decline-obesity relationship is complex with considerations, of type of memory, behavioral task and dietary intervention (fat, fat and sugar, sugar, and cafeteria diets) all adding to our overall understanding.

## Introduction

The rapid rise of obesity rates has been attributed to the increasing availability of unhealthy diets (that is over-consumption of food and beverages with a high content of fats, sugars and salt) and physical inactivity (WHO, 2015). The presence of overweight and obesity contributes to significant health impairments with large increases in the risk of cardiovascular disease, type 2 diabetes and cancer (McGee, 2005; Adams et al., 2006). The incidences of mild cognitive impairment (Elias et al., 2005; Jeong et al., 2005; Hassing et al., 2010), dementia (Whitmer et al., 2005; Anstey et al., 2011), and Alzheimer's disease (Solfrizzi et al., 2004; Whitmer et al., 2005; Gustafson et al., 2012; Besser et al., 2014) are similarly increased with obesity.

Rats fed high fat diets have been shown in some studies to be cognitively impaired compared to those fed a normal chow diet. Much emphasis has been placed on hippocampal-dependent behavioral tasks (Molteni et al., 2002; Wu et al., 2003; Goldbart et al., 2006; Pathan et al., 2008; Stranahan et al., 2008; Xia et al., 2015). In the Morris water maze (MWM), a number of studies have shown that high fat fed animals took longer to learn the location of a submerged platform relative to their control counterparts (Wu et al., 2003; Molteni et al., 2002; Goldbart et al., 2006; Pathan et al., 2008; Stranahan et al., 2008; Xia et al., 2015). These studies used varying levels of fat ranging from 21 to 58 kcal% and different lengths of diet consumption, with the general consensus that high fat diets consumed long-term can impair spatial learning and memory in the MWM.

The western diet (WD) model of obesity is a subtype of HFD-induced obesity that mimics the so-called “Western” diet by feeding rats a WD chow (containing 22% w/w fat equivalent to 40 kcal% fat) or a control chow diet (containing 6% total fat). The WD was formulated to represent a typical HFD typically consumed in developed “Western” countries and is equivalent to the *Harlan Teklad* TD88137 or *Research Diets* Western Diet D12079B that have previously been used to accelerate and enhance hypercholesterolemia and atherosclerotic plaque formation (Febbraio et al., 2000; Ascencio et al., 2004; Yang et al., 2006). We have previously shown that 12 week WD feeding causes a significant change in metabolic measures including increasing body weight, blood pressure and serum triglycerides (Kosari et al., 2012).

Dopamine (DA) has a well-recognized role in cognition including motivation, reward, punishment, and working memory (Cools, 2008). Recent research has discovered the involvement of DA with obesity (Volkow et al., 2012, 2013). It has been postulated that in individuals with a hypo-responsive mesocorticolimbic pathway there is an increased risk of the development of obesity (Davis et al., 2004). In humans sensitivity to reward has been associated with emotional overeating, preference for high fat foods, binge eating and food cravings (Loxton and Dawe, 2001; Davis et al., 2004, 2007; Franken and Muris, 2005). In a rodent study lentivirus-mediated knockdown of striatal DA D2-receptors resulted in the onset of compulsive-like food seeking in rats with extended access to palatable high-fat food and also decreased responsiveness of the brain reward system, consist with some of the evidence from humans (Johnson and Kenny, 2010). Moreover, a high fat cafeteria style diet also lowers both basal levels of DA and DA release in response to food or amphetamine (Geiger et al., 2009).

Given that we see a cognitive deficit in our animals after WD consumption (Kosari et al., 2012), along with important markers observed with weight gain and metabolic syndrome (Kosari et al., 2012), and the reported involvement of DA in obesity and reward, we hypothesized that the WD diet would produce modifications to the brain dopaminergic system and that this might lead to cognitive deficits. In this investigation we therefore examined cognitive function using the delayed-win shift (DWSh) task in the radial arm maze (RAM) (Jarrard, 1993; Floresco et al., 1997) in adult rats made obese from consuming the WD. This requires rodents to hold spatial information for food reward location during task performance, and across a delay (Seamans et al., 1995). Similar to the MWM, in that the rodent acquires, retains and uses trial-unique information, the DWSh task exploits food reward as a motivator. As such it can be suggested to be less stressful when compared to the MWM which relies on the rodents' swimming ability (Seamans et al., 1995). Furthermore, the task has a reliance on DA-rich regions, such as the medial prefrontal cortex (PFC) (Taylor et al., 2003), hippocampus (HPC) (Jarrard, 1993), and ventral striatum (Floresco et al., 1997; Jarrard, 1993). We then examined the effects of WD on neuronal activation within these regions along with DA content to assess how WD impacts on the ability to modulate these learning-associated brain regions to facilitate memory.

## Materials and methods

### Animals

Male Wistar hooded rats (University of Adelaide, Australia) were housed at RMIT University animal facility, a controlled environment (20 ± 1°C) with 12-h light/dark cycle (lights on at 07:00 h) in groups of 4, with food and water *ad libutum* in the home cage. Behavioral tests were performed from 9:00 to 19:00 h in a dedicated animal behavior room. All experiments were performed in accordance with the Prevention of Cruelty to Animals Act 1986 and with approval from the RMIT University Animal Ethics Committee.

### Dietary manipulation

Upon delivery, all animals were allowed to acclimatize for at least 1 week before commencement of dietary manipulation. Rats were randomly assigned to either a control diet (CON, Standard AIN93G rodent diet, 6% total fat including 1.05% total saturated fatty acids; Specialty Feeds, Perth, Australia) or WD (SF00-219, 21% total fat including 1.80% total saturated fats and 0.15% cholesterol; Specialty Feeds, Perth, Australia) and remained on this diet for 12 weeks.

## Experiment 1

### Food restriction

One week prior to the start of DWSh task, rats (*N* = 10 per group) were food restricted with their respective CON or WD. Body weight was monitored twice weekly to ensure rats do not fall below 85% of their free-feeding weight. Food restriction was maintained for the entire duration of behavioral testing.

### Delayed win-shift task in the radial arm maze

Testing was carried out in an eight-arm radial maze (Lafayette Instrument, USA), consisting of an octagonal central platform (34 cm diameter) and eight equally spaced radial arms (87 cm long, 10 cm wide). At the end of each arm was a food well (2 cm in diameter and 0.5 cm deep). At the start of each arm was a clear Perspex door that controlled access in and out of the central area. Each door was controlled by a computerized control box enabling the experimenter to control access to the arms. Salient visual cues of different geometric shapes were placed around the maze on the walls of the room.

On the first 3 days of testing, rats were habituated to the RAM in two sessions per day lasting 10 min each. After the final habituation session of the day, rats were returned to their home cages and given approximately 20 grain reward pellets (45 mg, Bio-Serv, USA). Following habituation, rats underwent a total of 12 training sessions with 2 sessions performed per day. This consisted of a 5 min training phase, 5 min inter-trial interval where the rat was returned to the home cage and a 5 min test phase. Before the training phase, 4 arms were pseudo-randomly chosen and blocked, with the following rule that no more than 2 adjacent arms could be closed in any trial. The remaining arms that were not blocked were baited with grain reward pellets. The training phase involved allowing the rat 5 min to enter and retrieve the grain pellet rewards from all the baited arms. After a 5 min inter-trial interval, the test phase occurred where all 8 arms were opened and the previously blocked arms are baited with grain reward pellets. The rat was then placed back inside the maze and the number of arm entries was recorded.

For analysis purposes, 2 training/test sessions were grouped into a single block. An arm entry was recorded when the animal fully moved off the central platform into the arm. Two types of errors were recorded: within phase error (working memory error, re-entry of an arm that has been baited and has been visited) and across phase error (reference memory error, entry into a training phase baited arm).

### Removal of epididymal adipose tissue

Once rats were culled with pentobarbital sodium (1 mg/kg), epididymal adipose tissue located within 10 mm from the epididymis (proximal) and within 10 mm from the distal end of the epididymal fat depot (distal) were harvested and weighed.

### HPLC sample preparation

Randomly selected rats from the RAM cohort (*N* = 5 per group) were killed by 0.5 ml i.p. injection of pentobarbital sodium (1 mg/kg). Brains were snap frozen in iso-pentane cooled to −35°C by dry ice then stored at −80°C. Whole striata, hippocampi and prefrontal cortices were dissected on ice with the use of the Paxinos and Watson rat brain atlas (Paxinos and Watson, 2007).

Prefrontal cortices, striata and hippocampi were assessed for DA and dihydroxyphenylacetic acid (DOPAC; DA metabolite) levels. Samples were homogenized in extraction buffer (4 M perchloric acid, 0.008 M sodium metabisulphate, 0.002 M disodium ethylenediaminetetra-acetic acid (EDTA) and MilliQ water to bring volume to 100 ml) and sonicated to rupture vesicular membranes. Samples were then spun at 10,500 g for 5 min, and the supernatant transferred to a fresh tube. The samples were spun a further two times, to ensure all debris was eliminated. Samples were stored at −80°C until required.

Forty μl of sample was transferred to a HPLC recovery vial. Standards for DA and DOPAC were made in the same extraction buffer used for sample preparation. The mobile phase was composed of 70 mM monopotassium phosphate, 0.5 mM EDTA disodium salt, 8 mM octane sulfonic acid sodium salt, 170 ml HPLC grade methanol, to a final volume of 1000 ml and pH 3. The flow rate was 500 μl/min with reverse phase C18 columns. HPLC analysis was conducted on PFC, striatal and HPC samples from rats fed either CON diet or WD for 12 weeks for total (intracellular and extracellular) DA and DOPAC levels. Standards of known concentrations for dopamine and DOPAC were used to quantify and identify the peaks on the chromatographs.

## Experiment 2

### Exposure to novel environment

A separate cohort of rats (*N* = 6–7 per group, 12 weeks CON or WD dietary manipulation) was assessed for activated Fos expression. Rats were placed into a novel arena, in our case a Y-maze (three-arm maze with equal angles between all arms which were 50 cm long × 17 cm wide × 32 cm high. Rats were allowed to move around this novel environment for 30 min. Rats were then returned to their home cages for 90 min in a dark, quiet room. This manipulation was to reduce exposure to other stimuli that might evoke Fos production. Immediately after this 90 min quiet period rats were deeply anesthetized with pentobarbitone sodium (1 mg/kg) and perfused transcardially with 0.1 M PBS followed by 4% paraformaldehyde in 0.1 M phosphate buffered saline (PBS).

### Home cage controls

In a further cohort of rats (*N* = 6 per group), underwent the identical dietary manipulation as the cohort above. These home cage control rats, run at a different time to the cohort above, remained untouched until culled when they were also deeply anesthetized with pentobarbital sodium (1 mg/kg) and perfused transcardially with 0.1 M PBS followed by 4% paraformaldehyde in 0.1 M PBS.

### Brain preparation

After transcardial perfusion heads were removed with a purpose built rat guillotine and brains removed and postfixed for 4 h in the 4% paraformaldehyde in PBS before placing them in 30% sucrose in PBS solution (4°C) until sectioning. Following fixing of brains, serial coronal sections (30 μm) were cut on a cryostat (Leica CM1950, Leica Microsystems, Germany) at −16°C and placed in cyroprotectant [30% (w/v) sucrose, 30% (w/v) ethylene glycol, 0.01% (w/v) polyvinyl pyrolidine in 0.1 M PBS (pH 7.4) solution] and stored at −20°C to later undergo immunohistochemistry.

### Fos immunohistochemistry

Sections were washed and transferred to 0.3% hydrogen peroxide in 0.1 M PBS containing 0.2% Triton X-100 (PBST) for 10 min to inhibit endogenous peroxidase and then washed several times with PBST. Sections were incubated in PBST containing Fos rabbit polyclonal antibody (1:5000; Ab-5; Oncogene Science, UK) for 48 h at 4°C with periodic rotation. Sections were then washed with PBST and incubated in biotinylated goat anti-rabbit secondary antibody (diluted 1:200 in PBST; Vectastain; Vector Laboratories, USA) and 1.5% normal goat serum for 2 h at room temperature on a rotator. Sections were then washed and processed with avidin-biotinylated horseradish peroxidase complex in PBST (Elite Kit; Vector Laboratories, USA) for 1 h at room temperature, again with constant rotation. Sections were washed again in PBST and then in 0.05 M Tris buffer. The reaction was then visualized using 3′, 3′–diaminobenzidine intensified with nickel chloride. Sections were mounted and allowed to dry overnight before being dehydrated via a graded series of alcohol washes and coverslipped.

### Image analysis

Photomicrographs of immunolabelled brain sections were captured at 10x objective using a BX60 microscope (Olympus, Japan) and RTKE SPOT camera (Diagnostic Instruments, USA) interfaced to a PC computer with SPOT imaging software. Counts of stained nuclei were carried out using the public domain Image J program (National Institutes of Health, USA). Images were digitized into gray scale where a threshold, set above the mean value ± S.E.M. of the background, was applied for background correction. Inside each region, the number of particles above the threshold was automatically calculated. There were no observed rostrocaudal differences in all brain regions analyzed.

### Regions of interest

A total of 7 regions were analyzed with sites selected because they have been implicated previously in memory processes. All of the sites from which it was decided *a priori* to count Fos-positive cells are presented. For each brain region analyzed, counts were taken from a minimum of four alternate coronal sections. Cytoarchitectonic subfields within the hippocampal formation consisting of the cornu ammonis area 1 (CA1), cornu ammonis area 2/3 (CA2/3) and dentate gyrus (DG) of the HPC were investigated. Hippocampal counts were taken at interaural 5.28 mm and bregma −3.72 mm in Paxinos and Watson rat brain atlas (Paxinos and Watson, 2007). Fos immunoreactive cells were counted in the prelimbic area (PrL), cingulate cortex (Cg), and infralimbic cortex (IL) corresponding to interaural 12.00 mm and bregma 3.00 mm (Paxinos and Watson, 2007). The striatum were counted at levels corresponding to interaural 11.04 mm and bregma 2.04 mm (Paxinos and Watson, 2007). Three areas within each striatal section were sampled using a 1 × 1 cm square generated the imaging program and a single value was obtained by averaging the 3 counts.

### Statistical analysis

All data are presented as mean ± S.E.M. A *p*-value of < 0.05 was considered statistically significant. Statistical comparisons were made between groups by repeated measures two-way ANOVA for DWSh task performance using GraphPad Prism version 6.00 (GraphPad Software, USA).

Two-way ANOVA was used for comparing HPLC data (neurotransmitter level x group). Basal and activated Fos counts were analyzed separately. Both the PFC and HPC counts were analyzed by two way ANOVA (subregion × group). Unpaired *t*-tests assessed basal and activated Fos counts separately for the striatum data. Further analysis by a *post-hoc* Bonferroni's *t*-test was performed if a significant effect was detected by the ANOVA.

## Results

### Effect of diet on metabolic measures

Rats fed a WD were observed to be heavier than rats fed a CON diet [Week 12, CON: 415.7 ± 9.8 g; WD: 458.2 ± 11.6 g; *F*_(1, 22)_ = 7.2 *p* < 0.05]. Both groups increased weight over time [time: *F*_(12, 264)_ = 0332.7, *p* < 0.0001] while WD rats increased their body weight at a more pronounced rate than CON, [group × time: *F*_(12, 264)_ = 3.5, *p* < 0.01]. *Post-hoc* analysis showed significant body weight differences starting from week 8 until week 12. WD consumption was shown to increase epididymal adipose tissue weight (CON: 8.2 ± 0.3 g; WD: 11.5 ± 0.6 g, *p* < 0.001).

### Experiment 1-radial arm maze and neurotransmitter changes

#### Training phase performance in the delayed win-shift task

**Figure 1:**
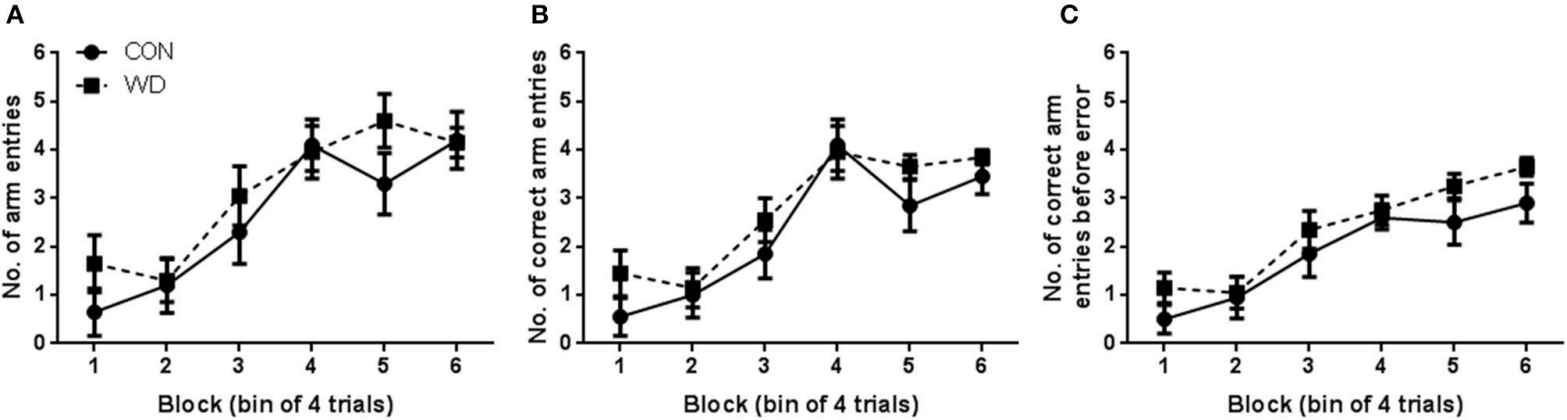
**Performance in training phase of DWSh task. (A)** Number of arm entries in each session of training. **(B)** Number of correct arm entries in each session of training. **(C)** Number of correct arm entries before error in each session of training. *n* = 10 per group.

Both CON and WD groups learnt to complete the DWSh task in the RAM, had fewer errors and entered more correct arms before an error was recorded as training progressed. Performances of rats in the training phase of the delayed win-shift radial arm maze procedure are shown in Figure 1. A repeated measures ANOVA was conducted and revealed that both CON and WD rats entered more arms as training progressed [*F*_(5, 90)_ = 29.86, *p* < 0.0001], however there was no group [*F*_(1, 18)_ = 0.63, *p* = 0.44] nor group x block effect [*F*_(5, 90)_ = 1.40, *p* = 0.23; Figure 1A]. During training, rats steadily increased the number of correct arm choices over blocks [block effect: *F*_(5, 90)_ = 36.34, *p* < 0.0001] but no group or group x block effect was observed (both *F* < 1, Figure 1B). As training progressed both CON and WD animals became more proficient in the task as the animals made more correct arm choices before an error was recorded [block effect: *F*_(5, 90)_ = 27.78, *p* < 0.0001; Figure 1C].

#### Test phase performance in the delayed win-shift task

**Figure 2:**
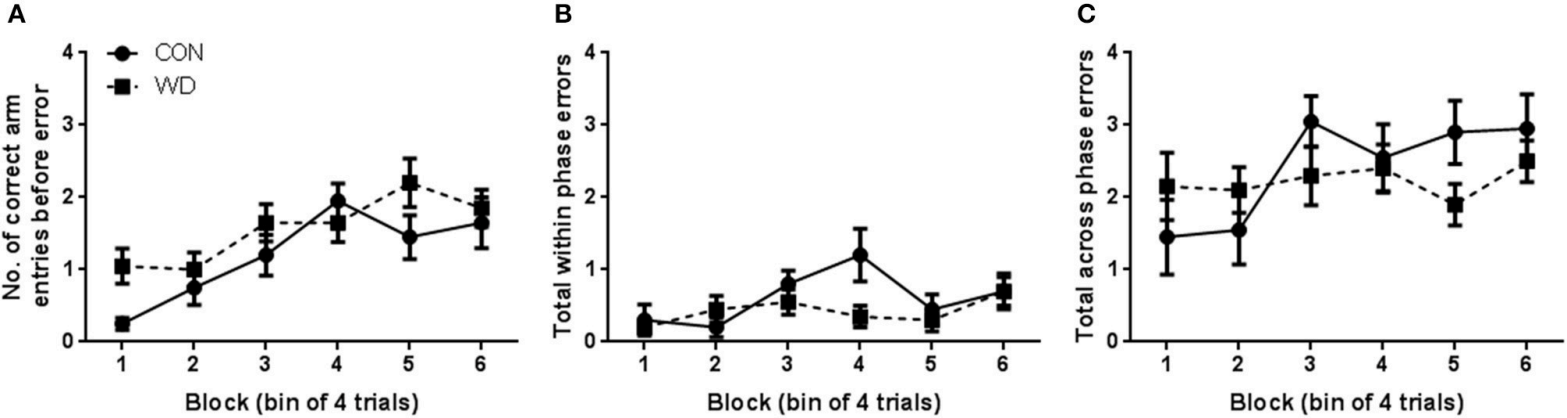
**Performance in test phase of DWSh task. (A)** Number of correct arm choices before error in each session of training. **(B)** Total number of within phase errors in each session of training. **(C)** Number of across phase errors in each session of training. *n* = 10 per group.

During the test phase, WD animals did not show any evidence of cognitive impairment relative to CON. The number of correct arm choices before an error was made steadily increased as training progressed [block effect: *F*_(5, 90)_ = 10.41, *p* < 0.0001; Figure 2A] and there was an overall effect of block to influence total within phase errors [*F*_(5, 90)_ = 2.79, *p* = 0.02; Figure 2B] but there was no other significant differences in any other measure including group (Figure 2C).

#### HPLC neurotransmitter analysis

**Figure 3:**
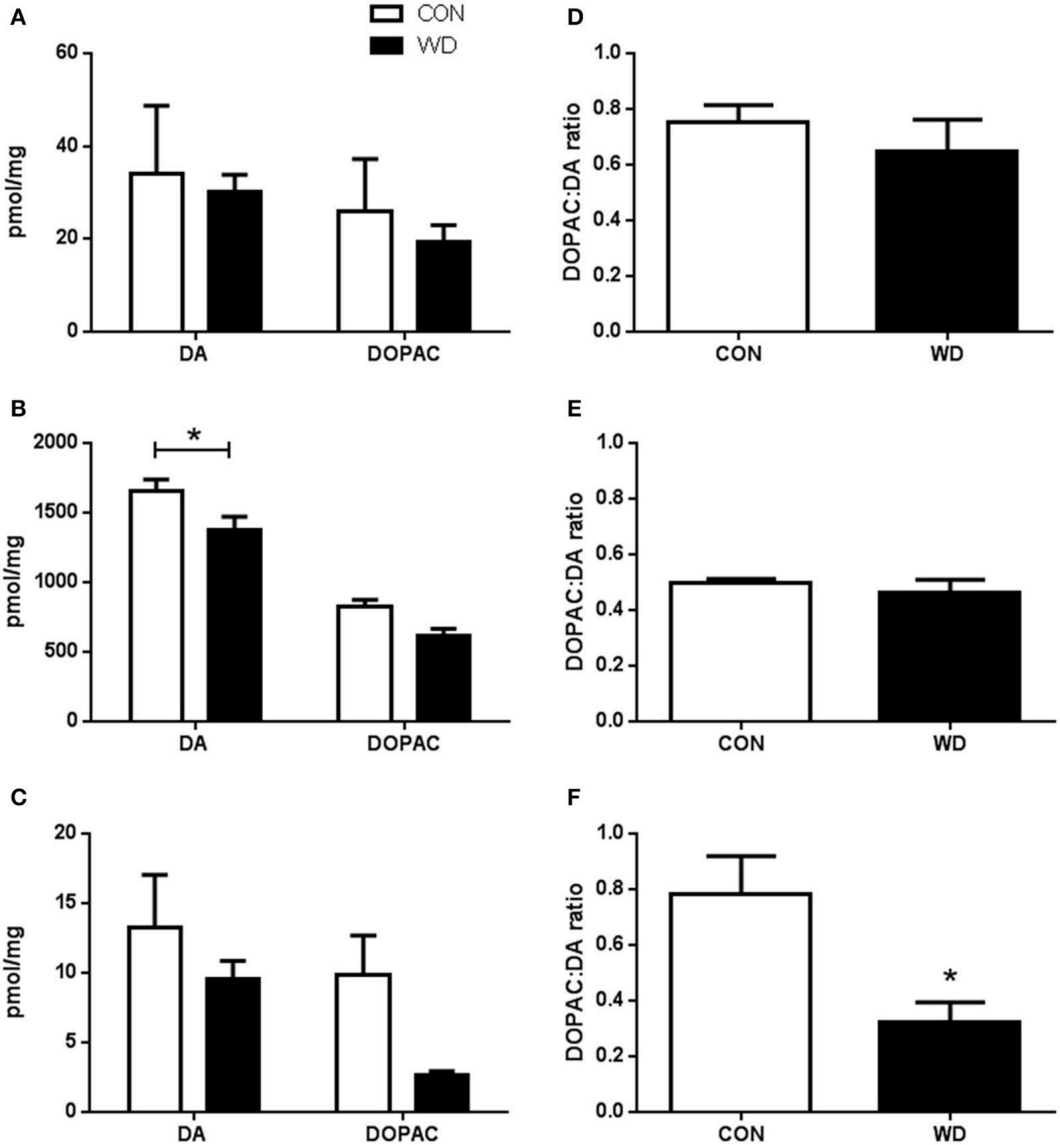
**HPLC analysis determination of DA, DOPAC, and DA turnover in rats fed a WD compared to CON in the (A)** prefrontal cortex. **(B)** striatum and **(C)** hippocampus. **(D–F)** HPLC analysis of DOPAC to DA ratio in the prefrontal cortex, striatum and hippocampus, respectively. *n* = 5 per group. ^*^Significantly different to CON *p* < 0.05.

In the PFC, WD consumption did not alter neurotransmitter levels (DA CON 34.01 ± 14.66 pmol/mg vs. WD 30.17 ± 3.66 pmol/mg and DOPAC CON 25.97 ± 11.24 pmol/mg vs. WD 19.43 ± 3.55 pmol/mg, *p* > 0.05; Figure 3A). No group or group x neurotransmitter effect was observed (all *F* < 1). DA turnover was also found not to be different between diet groups (CON 0.75 ± 0.06 vs. WD 0.65 ± 0.11, *p* > 0.05; Figure 3D).

WD feeding was observed to change DA and DOPAC levels in the striatum relative to CON with a significant effect of group [*F*_(1, 16)_ = 10.63, *p* = 0.0049], differing amounts of neurotransmitter [*F*_(1, 16)_ = 112.1, *p* < 0.0001] which was attributable to a significantly higher amount of DA than DOPAC (DA CON 1651.59 ± 85.61 pmol/mg vs. WD 1370.88 ± 99.45 pmol/mg; DOPAC (CON 822.54 ± 52.02 pmol/mg vs. WD 615.23 ± 49.83 pmol/mg) but not group x neurotransmitter interaction (*F* < 1). *Post-hoc* analysis showed a marked reduction in DA levels in the striatum relative to CON (*p* < 0.05; Figure 3B), but not DOPAC levels (*p* > 0.05). The DA turnover rate in the striatum was not seen to be influenced by WD consumption (CON 0.50 ± 0.02 vs. WD 0.46 ± 0.05, *p* > 0.05; Figure 3E).

In the HPC no differences were observed in neurotransmitter levels [*F*_(1, 16)_ = 4.3, *p* = 0.06], nor group x neurotransmitter (F < 1), while overall group effect was observed [*F*_(1, 16)_ = 4.9, *p* = 0.04]. However, with *post-hoc* analysis no individual differences were seen with DA (CON 13.22 ± 3.81 pmol/mg vs. WD 9.51 ± 1.34 pmol/mg, *p* > 0.05) or DOPAC (CON 9.88 ± 2.81 pmol/mg vs. WD 2.68 ± 0.27 pmol/mg, *p* > 0.05) levels compared to CON (Figure 3C). WD animals did have significantly reduced DA turnover relative to control (CON 0.78 ± 0.14 vs. WD 0.32 ± 0.07, *p* < 0.05; Figure 3F).

### Experiment 2-Fos immunohistochemistry

#### Home cage control Fos counts

Table 1 shows the expression of basal Fos in the regions analyzed after 12 week WD consumption. In the PFC subregions a two way ANOVA showed there was no effect of diet on home cage control Fos expression in the Cg, IL, and PrL regions group [*F*_(1, 30)_ = 0.95, *p* = 0.34]. Analysis of home cage control Fos in the striatum revealed no significant effect of diet (*p* = 0.59). The initial analysis of the HPC involved separate counts taken across subfields (CA1, CA2/3, and DG). Overall, there was no observed difference in home cage control Fos expression (group effect: [*F*_(1, 30)_ < 01, *p* = 0.99].

**Table 1:** **Number of positively stained Fos immunoreactive cells of home cage control fed CON or WD**.

**BRAIN REGION**	**CON (***n*** = 6)**	**WD (***n*** = 6)**
**PREFRONTAL CORTEX**		
Cingulate gyrus (Cg)	12.04 ± 2.03	12.83 ± 0.82
Infralimbic cortex (IL)	17.92 ± 2.77	15.46 ± 2.11
Prelimbic cortex (PrL)	18.88 ± 4.59	16.63 ± 1.86
**STRIATAL REGION**		
Striatum	117.50 ± 17.84	102.50 ± 19.78
**HIPPOCAMPUS**		
Cornu Ammonis area 1 (CA1)	61.70 ± 12.86	57.79 ± 19.01
Cornu Ammonis area 2/3 (CA2/3)	209.95 ± 55.71	196.13 ± 48.25
Dentate gyrus (DG)	116.70 ± 22.26	134.25 ± 44.27

#### Activated Fos counts

**Figure 4:**
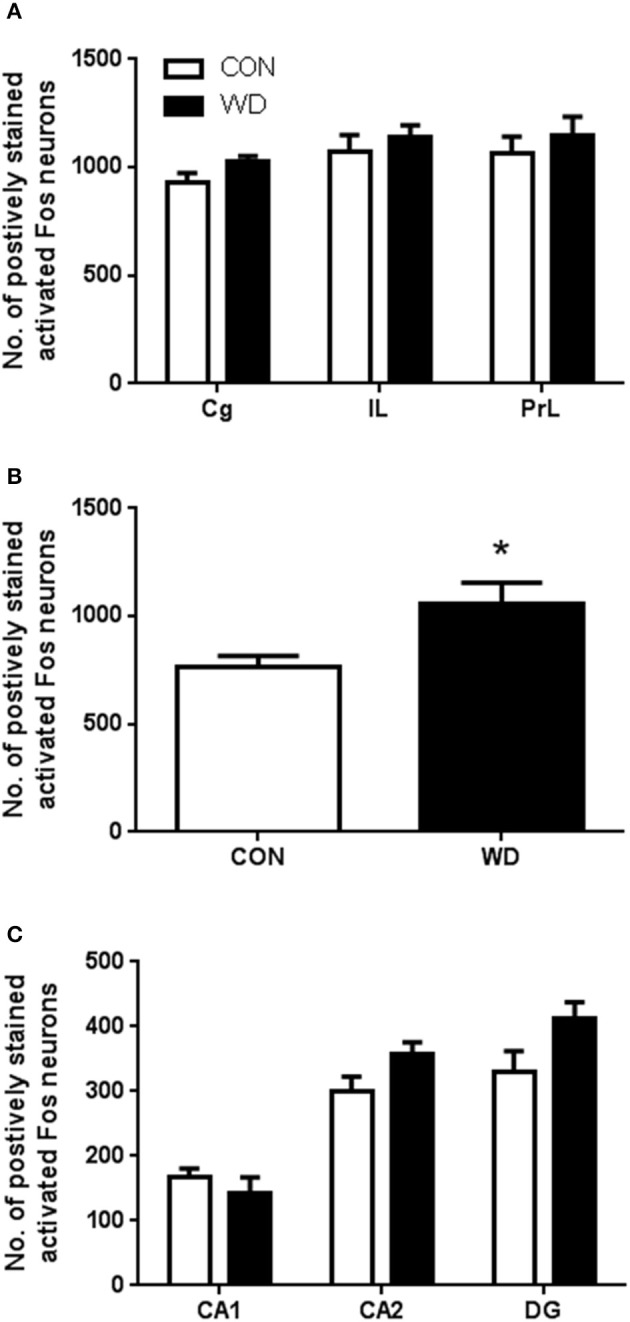
**Number of positively stained activated Fos neurons in the (A)** prefrontal cortex, **(B)** striatum and **(C)** hippocampus. *n* = 6-7 per group. ^*^Significantly different to CON *p* < 0.05.

Expression of activated Fos following novel environment stimulation in our CON and WD was examined. In the PFC subregions there was no effect of diet on activated Fos expression in the Cg, IL, and PrL regions group [*F*_(1, 33)_ = 2.36, *p* = 0.13; Figure 4A]. Analysis of stimulated neuronal activation in the striatum demonstrated a significant increase in Fos expression in this region in the WD-fed rats compared to controls (p < 0.05; Figure 4B). In the HPC subfields (CA1, CA2/3, and DG), there was no observed difference in activated Fos expression [group effect: *F*_(1, 30)_ = 3.67, *p* = 0.65; Figure 4C].

## Discussion

In this study we investigated the effect of WD on cognitive performance using the DWSh task in the RAM in adult male Wistar hooded rats. Our findings indicate that WD consumption caused a significant increase in body weight and epididymal adipose weight compared to controls, but did not affect learning and performance in the DWSh task, a PFC, HPC, and striatal-dependent memory task. Concurrently, there was an observed decrease in DA levels in the striatum and a reduction of DA turnover in the HPC in WD fed rats. In a further series of animals WD consumption was observed to be associated with an increase in activated Fos expression in the striatum after exposure to a novel environment.

Using the DWSh task, we failed to detect any impairment after WD consumption. Similar acquisition rates and spatial memory ability were observed in WD rats as the CON rats. This is in contrast to our previous study with this dietary manipulation where we showed impairment in spatial memory in the Y-maze, a one trial-one test procedure (Kosari et al., 2012). In the MWM, female Fisher 344 rats fed a diet with higher fat (approximately 39 kcal%) and similar sugar content to that in our study for 2 months displayed an impairment of spatial reference memory (Molteni et al., 2002). The radial arm water maze is known for combining the simplicity of results analysis from the RAM with the rapid and strong motivation observed in the MWM without the food deprivation (Alamed et al., 2006). Using the radial arm water maze, Alzhoubi et al. illustrated that male Wistar rats fed a similar WD for 3 months produced an impairment of short and long term spatial memory (Alzoubi et al., 2013). Researchers have also reported impairments of spatial memory following high fat consumption using other one trial-one test behavioral tasks. Arnold et al. reported spatial memory impairments using the T-maze spontaneous alternation task in C57BL/6J mice fed 45 kcal% fat diet for 8 weeks (Arnold et al., 2014), whilst a 60 kcal% fat diet for 27 weeks, produced an impairment of spatial reference memory in the object location task (Heyward et al., 2012). Of note between these studies, where a memory deficit was observed, and our present one, is a variation in food. Our DWSh paradigm prompts the animals to solve the task using food pellets as a reward, and for both WD and CON animals we used the same “control” grain pellets. A consequence of this is that WD animals, if they solved the task successfully, consumed approximately sixteen 45 mg pellets/day during habituation and testing that were not of WD composition. Moreover, throughout the task animals were on food restriction, albeit of their WD or CON food, meaning that for the final 15 days all rats received less of their specific diet than the previous 12 weeks of *ad lib* feeding. These two methodological points may have resulted in our WD animals becoming normalized, and thus attributable to the lack of deficit in this task.

We demonstrated that 12 week WD consumption increased activated Fos expression in the striatum in response to a novel environment. Other brain regions involved in memory and learning were also investigated with no comparable differences in activated Fos expression in the PFC or HPC between control and WD animals. C57BL/6J mice fed a 58 kcal% fat diet for 15 weeks display increased expression of basal Fos in the lateral hypothalamus (Lin and Huang, 1999; Xin et al., 2000), dorsal medial hypothalamus (Xin et al., 2000; Lin and Huang, 1999), and paraventricular nuclei (Wang et al., 1999). A study in female Long-Evans rats fed a 40 kcal% fat diet for 12 weeks also demonstrated an increase of activated Fos expression in the paraventricular nuclei induced by introduction into a novel environment (Ressler et al., 2015). In a further study, acute high fat diet (21 kcal% fat for 2 h) consumption in C57BL/6J mice elicited an increase of activated Fos expression not only in the lateral hypothalamus but also the ventral tegmental area, nucleus accumbens, and central amygdala (Valdivia et al., 2014), suggesting that acute high fat consumption recruits the mesolimbic system. This finding is corroborated by Del Rio et al. who demonstrated that the dorsal medial PFC selectively showed increased activated Fos immunoreactivity in response to acute high fat diet consumption (Del Rio et al., 2015).

Whilst the stimulus used to induce activation of Fos expression was the introduction of the animal into a novel environment, our group has not observed any differences of exploration time in novel environments with this model (Kosari et al., 2012). We would suggest that this indicates that the increase of neuronal activation is due to the diet manipulation and not the stimulus, in this case new surroundings. However, it should be considered that there may also be an interaction between the experience of stress and the western diet. In the water maze RAM study (Alzoubi et al., 2013), the combination of stress and western diet resulted in the strongest impairment in the memory test, suggesting that stress may exacerbate the effect of diet.

The presented results also demonstrate that WD consumption for 12 weeks causes a dysregulation of the dopaminergic system in the striatum and HPC, as reflected by the decrease of DA levels in the striatum by 20% and DA turnover in the HPC by 40% in WD rats compared to controls. Our findings parallel data observed by Ma et al. who also showed a decrease of DA levels and no change in DA turnover in the striatum of rats fed a 60 kcal% HFD for 13 weeks (Ma et al., 2015). Results by Baladi et al. using chronoamperometry also indicated a decrease in DA turnover in the striatum though interestingly this was independent to any observed changes to body weight in high fat diet fed rats (Baladi et al., 2015).

Multiple studies have also shown that obese animals have a decrease of dopaminergic levels in other limbic areas of the brain and/or decreased DA receptor expression. A consistent finding is the reduction of DA levels in the nucleus accumbens in animal models of obesity observed in *ob/ob* mice (Fulton et al., 2017), diet-induced obesity rats (Pothos et al., 1998; Geiger et al., 2009), cafeteria diet model (Geiger et al., 2008) and high fat diet exposed mice (Carlin et al., 2013). Mice fed a 60 kcal% fat diet for 12 weeks also had a decrease of DA levels and increased DA turnover in the PFC (Carlin et al., 2013). High fat diet exposure, even as short as 5 days, has been shown to reduce basal DA levels in the nucleus accumbens (Rada et al., 2010). The reduction in DA levels at least in the nucleus accumbens is suggested to be due to reduced stimulated DA release and vesicle size (Pothos et al., 1998; Geiger et al., 2008). Additionally, DA reuptake has been observed to decrease independent of DA transporter protein gene expression in rats fed a high fat diet, thought to be due to interference in DA transporter trafficking or maturation (Petrovich et al., 2007).

Striatal DRD2 receptor expression has been previously shown to be decreased after consumption of a HFD in both mice and rat models of diet-induced obesity (Huang et al., 2006; Johnson and Kenny, 2010; van de Giessen et al., 2012). One study also found a clear inverse association between body weight and striatal DRD2 receptor expression suggested to be due to a down regulation of postsynaptic striatal DRD2/3 receptors (Huang et al., 2006; Johnson and Kenny, 2010; van de Giessen et al., 2012). Thus, it could be hypothesized that WD consumption alters DRD2 receptor expression which can lead to the neuroadaptive response to decrease DA levels in the striatum and DA turnover in the HPC. It is known that DRD2 receptor plays an inhibitory role in dopaminergic transmission in the mesolimbic dopaminergic system (Nestler, 1994). Previous observations have shown an inverse correlation between adiposity and striatal D2 binding in HFD fed mice (Huang et al., 2005), rats (Johnson and Kenny, 2010) and obese humans (Davis and Fox, 2008).

The mechanisms of how a WD, or in a broader context high fat diets, can produce memory impairments is still under much scrutiny. Our lab has previously considered the possibility that the cholinergic system is associated with spatial deficits caused by WD consumption (Kosari et al., 2012). Nonetheless using immunohistochemistry we reported no change in acetylcholinesterase activity, the enzyme responsible for the metabolism of acetylcholine, in the HPC and striatum after WD consumption (Marco et al., 2013). WD consumption was not observed to affect spatial working and reference memory using the DWSh task in the RAM. The present study is the first to demonstrate that WD consumption increases Fos expression in the striatum following a novel environment stimulus. We also show that WD consumption induces a reduction in DA levels in the striatum and DA turnover in the HPC. This suggests that WD consumption induces central changes in the striatum and HPC through neuronal activation which could be mediated through DA activity. However, these changes are independent to impairments in spatial working or reference memory in WD fed rats.

In conclusion our present results expand on the known relationship between obesity and central effects. We have shown that a major neurochemical component of cognition and reward, dopamine, is reduced after WD consumption in our rats, an effect that has been largely observed in rodents following a more palatable food intake, suggesting that the type of diet, that is sugary and appealing, is not specific to producing neurochemical changes in higher order brain regions. We have also extended the previous neuronal activation data largely focused around the hypothalamus to show that WD-manipulation in the rat produces specifically an upregulation of striatal neuronal activation. As this data was collected from a novel environment paradigm of much interest would be to expand this to assess neuronal activation during memory tests where a cognitive deficit is observed. Whether WD manipulation, or indeed fat manipulation, is the ideal model to assess obesity-associated cognitive decline is still contentious; indeed our Fos data, and the reduction in striatal dopamine content, is independent of a deficit in memory using the radial arm maze task. While we have previously shown cognitive deficits along with metabolic changes with this model (Kosari et al., 2012) it is not universal; indeed no deficit was observed in working memory in the novel object recognition task (Kosari et al., 2012), as no deficiency was observed here. It is clear that the biological contribution to obesity in humans involves numerous factors beyond fat, including the more palatable sugar, inactivity, along with broader factors, such as genes and mood, and these are yet to be considered or produced in a single animal model.

## Author contributions

JN with TJ, SK, SA, and OW were involved in the conception and design of the studies. Data collection was performed by JN, SA, and SK; Data analysis and interpretation by JN, SS, and TJ. The article was drafted by JN, ASK, and TJ with critical revision and final approval of the version to be published from all authors.

### Conflict of interest statement

The handling Editor declared a shared affiliation, though no other collaboration, with one of the authors SK, and states that the process nevertheless met the standards of a fair and objective review. The other authors declare that the research was conducted in the absence of any commercial or financial relationships that could be construed as a potential conflict of interest.
